# Glyphosate-Dependent Inhibition of Photosynthesis in Willow

**DOI:** 10.3389/fpls.2017.00207

**Published:** 2017-02-17

**Authors:** Marcelo P. Gomes, Sarah G. Le Manac’h, Louise Hénault-Ethier, Michel Labrecque, Marc Lucotte, Philippe Juneau

**Affiliations:** ^1^Ecotoxicology of Aquatic Microorganisms Laboratory, GRIL, TOXEN, Department of Biological Sciences, Université du Québec à Montréal, MontréalQC, Canada; ^2^Laboratório de Fisiologia Vegetal, Instituto de Ciências Biológicas, Departamento de Botânica, Universidade Federal de Minas GeraisBelo Horizonte, Brazil; ^3^Institut des Sciences de l’Environnement, Université du Québec à Montréal, MontréalQC, Canada; ^4^Institut de Recherche en Biologie Végétale, Montreal Botanical Garden, MontréalQC, Canada

**Keywords:** herbicide, oxidative stress, photosynthesis, proline, shikimate, willow

## Abstract

We studied the physiological mechanisms involved in the deleterious effects of a glyphosate-based herbicide (Factor^®^ 540) on photosynthesis and related physiological processes of willow (*Salix miyabeana* cultivar SX64) plants. Sixty-day-old plants grown under greenhouse conditions were sprayed with different rates (0, 1.4, 2.1, and 2.8 kg a.e ha^-1^) of the commercial glyphosate formulated salt Factor^®^ 540. Evaluations were performed at 0, 6, 24, 48, and 72 h after herbicide exposure. We established that the herbicide decreases chlorophyll, carotenoid and plastoquinone contents, and promotes changes in the photosynthetic apparatus leading to decreased photochemistry which results in hydrogen peroxide (H_2_O_2_) accumulation. H_2_O_2_ accumulation triggers proline production which can be associated with oxidative protection, NADP^+^ recovery and shikimate pathway stimulation. Ascorbate peroxidase and glutathione peroxidase appeared to be the main peroxidases involved in the H_2_O_2_ scavenging. In addition to promoting decreases of the activity of the antioxidant enzymes, the herbicide induced decreases in ascorbate pool. For the first time, a glyphosate-based herbicide mode of action interconnecting its effects on shikimate pathway, photosynthetic process and oxidative events in plants were presented.

## Introduction

Glyphosate [*N*-(phosphonomethyl)glycine)] is the most broadly used herbicide worldwide since the introduction of glyphosate-resistant (GR) plants (Coupe et al., 2012). Although it has been suggested as one of the least toxic pesticides to animals and humans (Williams et al., 2000; Cerdeira and Duke, 2006), the widespread use of glyphosate together with its great solubility trigger some concerns about its possible effects on the environment.

Glyphosate negative effects on non-target plants (Bott et al., 2011) and aquatic organisms (Vendrell et al., 2009; Inderjit and Kaushik, 2010) have been largely described. By inhibiting the EPSP synthase (EC 2.5.1.19), glyphosate-based herbicides prevent biosynthesis of aromatic amino acids (Siehl, 1997) leading to shikimic acid accumulation (Duke and Powles, 2008). The depletion of the aromatic amino acid pool leads to a reduction of protein synthesis necessary to growth maintenance (Siehl, 1997). On the other hand, in some plants, aromatic amino acid deficiencies upon glyphosate application has not been found, although deleterious effects of the herbicide have been observed (Lee, 1981; Wang, 2001; Serra et al., 2013). This indicates that glyphosate can affect other plant-physiological processes (Gomes et al., 2014b). Numerous studies demonstrated decreases in the photosynthetic rate of plants following treatment with glyphosate (Mateos-Naranjo et al., 2009; Yanniccari et al., 2012; Zobiole et al., 2012). In glyphosate-sensitive plants, the herbicide causes inhibition of CO_2_ assimilation (Diaz Vivancos et al., 2011) and depletion of intermediates of the photosynthetic carbon reduction cycle (Servaites et al., 1987) which could be linked to the unregulated flux of carbon into the shikimate pathway (Siehl, 1997). Moreover, glyphosate can indirectly affect photosynthesis by inhibiting chlorophyll biosynthesis (Fedtke and Duke, 2005) or inducing chlorophyll degradation (Gomes et al., 2016a), decreasing stomatal conductance (Yanniccari et al., 2012), and provoking nutritional disturbances (Cakmak et al., 2009; Su et al., 2009). Nowadays, a special attention has been giving to understand glyphosate-induced oxidative stress in plants (Gomes et al., 2014b).

Reactive oxygen species are essential in plant signaling; however, once accumulated, ROS become toxic, inducing irreversible changes in metabolism, cell cycle, and increase oxidative bursts (Gomes et al., 2014a). By interacting with biological molecules, ROS can induce destruction of DNA, lipids, and proteins (Foyer and Noctor, 2011). To avoid oxidative damage due to ROS accumulation, plants have developed enzymatic (e.g., SOD, CAT, APX, GPX, and GR) and non-enzymatic (e.g., ascorbate and glutathione) systems (Foyer and Noctor, 2011). The activity of antioxidant systems as well as the lipid peroxidation extent are oxidative stress markers which were shown to be modulated by glyphosate exposure (Ahsan et al., 2008; Moldes et al., 2008; Miteva et al., 2010).

Glyphosate effects on photosynthesis of non-resistant plants were associated to the herbicide induced decreases in the abundance of photosynthetic pathway proteins together with the oxidation of the major redox pools (Diaz Vivancos et al., 2011). However, it has been reported that glyphosate can also induce ROS accumulation (Ahsan et al., 2008; Moldes et al., 2008; Miteva et al., 2010; Gomes et al., 2016a) and glyphosate-resistance was related to the ability of plants to avoid oxidative bursts through activation of antioxidant systems (Maroli et al., 2015). Photosynthesis-targeting herbicides, such as atrazine, are known to induce oxidative stress by inhibiting Hill’s reactions (Fedtke and Duke, 2005). Plants exposed to these kinds of herbicides are not able to cope with the mass of triplet chlorophyll molecules produced due the blockage of the electron transport flow, resulting in cell oxidative bursts due to ROS accumulation. On the other hand, it is not clear how glyphosate can induce ROS accumulation in plants and if the oxidative stress induced by the herbicide could also be related to the observed decreases in photosynthesis. We hypothesized that the interference on shikimate pathway could induce ROS production and consequently affect photosynthesis of exposed plants. Therefore, in this study we accessed the physiological mechanisms involved in the deleterious effects of a glyphosate-based herbicide (Factor^®^ 540) on photosynthesis of willow (*Salix miyabeana* cultivar SX64) plants. For the first time, a glyphosate-based herbicide mode of action interconnecting glyphosate effects on shikimate pathway, plant photosynthetic process and oxidative events were described.

## Materials and Methods

### Greenhouse Experiments

*Salix miyabeana* cultivar SX64 was chosen for this study due to its high tolerance to stress factors, fast growth and great biomass production (Labrecque and Teodorescu, 2005). Moreover, this species has been indicated for phytoremediation programs, particularly in the context of riparian buffer strips, to reclaim agricultural contaminants (Gomes et al., 2016b). Cuttings of *S. miyabeana* approximately 20 cm long were grown in plastic boxes (35 l) filled with distilled water amended with King Max nutrient solutions A (7% P_2_O_5_, 11% K_2_O, 1.5% Mg, 1.27% S, 0.07% B, 0.002% Mo, 0.12% Zn) and B (4% N, 1% NH_4_^+^, 3% NO_3_^-2^, 10% K_2_O, 2% Ca, 0.05% Fe, 0.05% Mn) (Montreal, QC, Canada), following the product’s instructions. The solutions were continuously aerated, and renewed every 15 days. The pH of the medium was checked and adjusted on a weekly basis to 6.5 ± 0.1. The greenhouse was maintained at 25/22°C (±3°C) day/night temperature with natural light supplemented by sodium vapor lamps to provide a 12 h photoperiod and an average photosynthetic active radiation of 825 μmol photons m^-2^ s^-1^. After an initial growth period (45 days), rooted, healthy (without leaf chlorotic spots) and uniform (similar height) plants were used in all treatments. A randomized block design with seven containers (corresponding to the replicates) per treatment, in a 4 (herbicide concentrations) × 4 (times of evaluation) factorial scheme was used. One hundred microliters of a freshly prepared herbicide solutions were hand-sprayed uniformly on each of the first three fully expanded leaves (corresponding to seventh to ninth leaves counting down from the shoot apex). This spray volume did not result in any runoff from the leaves. The herbicide (0, 56.15, 84.21, and 112.30 mM of glyphosate) applied concentrations were equivalent to field applications of 0, 1.4, 2.1, and 2.8 kg glyphosate ha^-1^, which represent scenarios of 50, 75, and 100% of the standard field herbicide concentration applied in agricultural areas in Quebec (Gomes et al., 2016a).

Photosynthetic (using chlorophyll fluorescence kinetic measurements) and biochemical evaluations were performed at 0, 6, 24, 48, and 72 h after the beginning of the treatments. The evaluations were stopped after 72 h of exposure as plants from the highest glyphosate treatment showed pronounced intoxication symptoms, including several necrotic spots and loss of leaves (data not shown). After photosynthetic and stomatal conductance evaluations, plants were harvested and thoroughly washed with distilled water. Samples of the seventh (first fully expanded leaf from the apex) to ninth leaves were immediately frozen in liquid nitrogen and stored in aluminum foil paper at -80°C until biochemical evaluations and oxidative damage evaluations.

### Gas Exchange, Chlorophyll Fluorescence, and Pigment Concentrations

Gas exchange, chlorophyll fluorescence, and pigment contents were measured on samples from the first, second, and third fully expanded leaves (seventh–ninth leaves from the apex), which also received the herbicide, for a total of three measurements per plant. Measurements of stomatal conductance (*g*_s_) were performed using a leaf porometer (model SC-1, Decadon Devices Inc., Washington, DC, USA). Then, these leaves were dark-acclimated for 20 min and the chlorophyll fluorescence emission was assessed using a pulse-amplitude modulation (PAM) fluorometer (model PAM-2500, WALZ, Effeltrich, Germany). A RLC analysis was performed according to Juneau et al. (2015). An 11 steps RLC was performed. Saturating pulses were triggered at 0.8 min intervals with varying actinic light intensity for each step (0, 31, 48, 76, 117, 179, 253, 405, 586, 874, and 1326 μmol photons m^-2^ s^-1^). Using the RLC, the evaluation of the following parameters was performed: the ETR (Krall and Edwards, 1992), the qP (van Kooten and Snel, 1990), the UQF_rel_ (Juneau et al., 2005), the NPQ (Redondo-Gómez et al., 2008), and the *F*_V_/*F*_M_ (Kitajima and Butler, 1975). To compare treatments, fluorescence results from the 874 μmol photons m^-2^ s^-1^ (most similar irradiation in relation to light growth conditions) were used. Curves of ETR versus irradiance were also plotted and the ETR_max_ and the *I*_k_ were calculated according to Eilers and Peeters (1988).

For pigments evaluations, three foliar disks of approximately 5 mm in diameter were taken from each leaf, and after determining the fresh weight of the samples, their chlorophyll and carotenoid pigments were extracted in 80% acetone after macerating the disks with a mortar and pestle. The spectral absorption of the extracts (from 300 to 800 nm) was measured using a Varian Cary^®^ 300 Bio UV-Vis spectrophotometer (Varian, USA). The concentrations (μg/g fresh leaf weight) of total chlorophylls and carotenoids were calculated using the equations described by Lichtenthaler and Wellburn (1983).

### Biochemical Evaluations

Shikimate and proline concentrations were evaluated following the methods described in Bates et al. (1973) and Bijay and Dale (1998), respectively. To evaluate the pool of quinones in leaves, 0.1 g of fresh plant tissue was ground in liquid nitrogen, homogenized in 1000 μl of freeze-cold ethyl acetate and then centrifuged for 1 min at 6.590 × *g* (Kruk and Karpinski, 2006). The supernatant was then transferred to a collecting tube and the procedure was repeated twice (by adding 1000 μl of freeze-cold ethyl acetate to the pellet) to assure high extraction efficiency. Ten microliters of cold 1 M sodium borohydride (NaBH_4_) was added to the combined supernatant to convert quinone to its reduced form and then, samples were centrifuged for 2 min at 10.000 × *g* to remove impurities (Yoshida et al., 2010). The standard of plastoquinone (PQ-9, 1 mM) was acquired from the laboratory of J. Kruk (Jagiellonian University, Poland). After dilution in ethanol, the amount of 20 μl of cold 1 M NaBH_4_ was added to assure complete reduction of plastoquinone pool. The UHPLC (Agilent 1290 Infinity II LC, Wilmington, DE, USA) measurements were performed according to Yoshida et al. (2010), using UV-VIS detector, fluorescence detector, column (50 mm × 2.1 mm) isocratic solvent system (methanol/hexane, 340/20 vol/vol), flow rate of 0.31 ml/min, absorption detection wavelength at 255 nm, fluorescence excitation/emission detection at 290/330 nm, and injection volume of 1 μl.

To assess oxidative responses, H_2_O_2_, MDA contents and the activity of antioxidant systems were studied following the methods described by Gomes et al. (2014c). H_2_O_2_ was extracted in 2 ml of 0.1% trichloroacetic acid (TCA) and after centrifugation at 12000 × *g* for 15 min, 300 μl of the centrifuged supernatant was reacted with 0.5 ml of 10 mM potassium phosphate buffer (pH 7.0) and 1 ml of 1 M KI. Samples were read at 390 nm and H_2_O_2_ concentrations were determined using an extinction coefficient (𝜀) of 0.28 mM^-1^ cm^-1^. The estimation of lipid peroxidation was based on the production of 2-thiobarbituric acid reactive metabolites, particularly MDA. Samples containing 200 mg of leaf and root tissue were macerated in 5 mL of 0.1% TCA. After complete homogenization, 1.4 mL of the homogenate was transferred to an eppendorf tube and centrifuged at 10,000 rpm for 5 min. An aliquot of 0.5 mL of the supernatant was added to 2 mL 0.5% (v/v) TBA (thiobarbituric acid) in 20% TCA. The mixture was heated in a water bath at 95°C for 30 min and then ice-cooled for 10 min. Readings were taken using a spectrophotometer at 535 and 600 nm.

To study the antioxidant enzymes, 0.1 g of leaves were macerated in 800 μl of an extraction buffer containing 100 mM potassium buffer (pH 7.8), 100 mM EDTA, 1 mM L-ascorbic acid and 2% PVP (m/v). The protein contents of samples were determined using the Bradford method. Activities of SOD (EC 1.15.1.1), CAT (EC1.11.1.6), APX (EC 1.11.1.11), GPX (E.C. 1.11.1.9), and GR (E.C. 1.6.4.2) were assessed. To evaluate the ascorbate pool [total ascorbate (AsA + DHA), AsA and DHA], 0.2 g of frozen tissue were ground in liquid nitrogen in a mortar and pestle and homogenized with 5 ml of 6.5% (w/v) *m*-phosphoric acid containing 1 mM NaEDTA.

### Statistical Analyses

Results were expressed as the average of three replicates. Statistical analyses were performed using JMP software 10.0 (SAS Institute Inc). Results were submitted to normality (Shapiro–Wilk) and homogeneity (Bartlett) tests and then statistically evaluated. MANOVA univariate repeated measures, with Time as the within-subject factor and the herbicide concentrations as the main effects, were used to analyze differences in the variables studied during exposure to the treatments. Glyphosate, Time, and the interaction between glyphosate and time were included within the model. The sphericity of the data was tested by the Mauchly’s criteria to determine whether the univariate *F* tests for the within-subject effects were valid. In cases of invalid *F*, the Greenhouse–Geisser test was used to estimate epsilon (𝜀). Contrast analysis was used when there were significant differences in the variables between treatments (Supplementary Tables 1S and 2S).

## Results

### Pigment Content, Gas Exchange, and Chlorophyll Fluorescence

**FIGURE 1:**
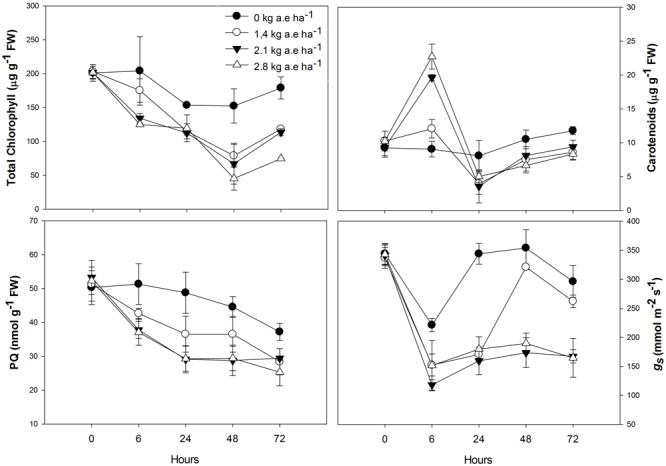
**Time courses of pigment (total chlorophyl and carotenoids) concentrations, plastoquinone pool (PQ), and stomatal conductance (*g*_s_) in leaves of *Salix miyabeana* (cultivar SX64) plants spread with doses of increased (0, 1.4, 2.1, and 2.8 kg a.e ha^-1^) rates of the glyphosate based herbicide (Factor^®^ 540).** Values are means ± SE of three replicates.

**FIGURE 2:**
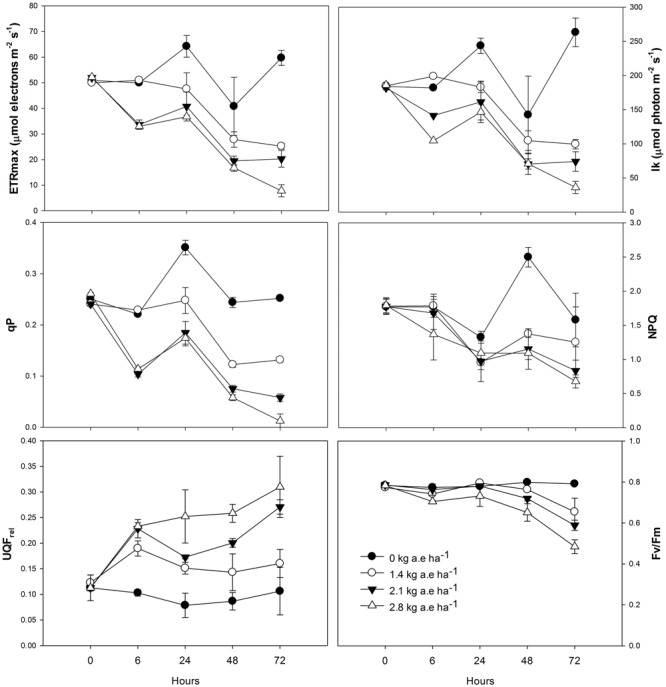
**Time courses of photosynthesis-related measurements [maximum electron transport rate (ETR_max_), minimum saturating irradiance (*I*_k_), photochemical quenching (qP), non-photochemical quenching (NPQ), relative unquenched fluorescence (UQF_rel_), and maximal photochemical efficiency of PSII (*F*_V_/*F*_M_)] in leaves of *Salix miyabeana* (cultivar SX64) plants spread with doses of increased (0, 1.4, 2.1, and 2.8 kg a.e ha^-1^) rates of the glyphosate based herbicide (Factor^®^ 540).** Values are means ± SE of three replicates.

Total chlorophyll and plastoquinone concentrations were decreased in leaves of plants by herbicide exposure and by treatment time (*P* > 0.001; **Figure 1**). The carotenoid concentration was greater in herbicide-treated plants at 6 h for all applied doses (**Figure 1**); then, carotenoid concentration was decreased in plants exposed for at least 24 h to herbicide concentrations (*P* < 0.0001). The stomatal conductance was decreased in herbicide-exposed plants for all the treatment times (*P* < 0.05; **Figure 1**). Similar effects were observed on the ETR_max_, the *I*_k_, and the qP, which were significantly reduced in treated plants (*P* < 0.0001; **Figure 2**). However, for the first evaluation (6 h), ETR_max_, *I*_k_, and qP were not decreased in plants treated with 1.4 kg a.e ha^-1^ (*P* > 0.05; **Figure 2**). The UQF_rel_ increased in all treated plants (**Figure 1**). Concomitantly, the NPQ decreased in plants exposed for more than 24 h to the herbicide (*P* < 0.05; **Figure 2**). The maximal PSII photochemical efficiency (*F*_V_/*F*_M_) was decreased in herbicide-treated plants (*P* < 0.0001). Decreased *F*_V_/*F*_M_ was seen in plants treated with 1.4 kg a.e ha^-1^ only after 72 h of herbicide exposure (*P* < 0.05; **Figure 2**). Plants exposed to 2.1 kg a.e ha^-1^ showed decreases in *F*_V_/*F*_M_ at 48 and 72 h of exposure (*P* < 0.001). In contrast, in all the evaluations, plants exposed to 2.8 kg a.e ha^-1^ showed decreased *F*_V_/*F*_M_ (*P* < 0.01; **Figure 2**).

### Shikimate and Proline Contents

**FIGURE 3:**
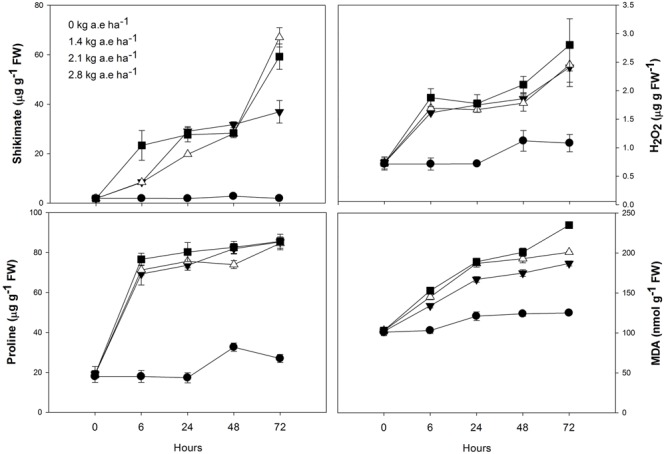
**Time courses of shikimate, proline and hydrogen peroxide concentrations, and lipid peroxidation (MDA concentrations) in leaves of *Salix miyabeana* (cultivar SX64) plants spread with doses of increased (0, 1.4, 2.1, and 2.8 kg a.e ha^-1^) rates of the glyphosate based herbicide (Factor^®^ 540).** Values are means ± SE of three replicates.

The shikimate and proline concentrations in leaves of herbicide-treated plants were always higher than the control (*P* < 0.0001; **Figure 3**). In plants exposed to 2.1 and 2.8 kg a.e ha^-1^, an important shikimate accumulation was found after 72 h of herbicide-treatment (*P* < 0.0001).

### H_2_O_2_ Contents and Lipid Peroxidation

Compared to control, H_2_O_2_ concentration was always higher in plants exposed to the herbicide (*P* < 0.001; **Figure 3**), and greatly increased in these plants after 72 h (*P* < 0.01). Similarly, lipid peroxidation (MDA concentration) was always higher in plants exposed to the herbicide (Supplementary Table 2S; **Figure 3**). In all plants, MDA content slightly increased at 24 h (*P* > 0.05). However, in plants treated with herbicide, a pronounced increase in MDA concentration was observed at 72 h (*P* < 0.05).

### Antioxidant Responses

**FIGURE 4:**
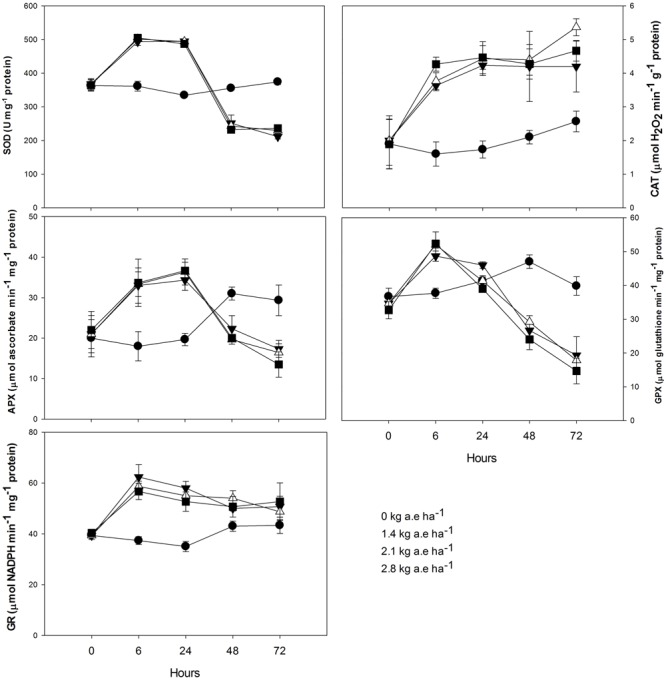
**Time courses of superoxide dismutase (SOD), catalase (CAT), ascorbate peroxidase (APX), glutathione peroxidase (GPX), and glutathione reductase (GR) activities in leaves of *Salix miyabeana* (cultivar SX64) plants spread with doses of increased (0, 1.4, 2.1, and 2.8 kg a.e ha^-1^) rates of the glyphosate based herbicide (Factor^®^ 540).** Values are means ± SE of three replicates.

**FIGURE 5:**
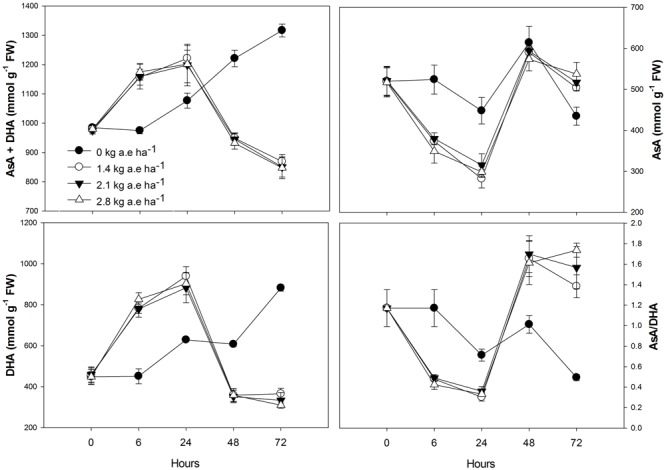
**Time courses of total ascorbate (AsA + DHA), reduced ascorbate (AsA), oxidized ascorbate (DHA), and AsA/DHA ratio in leaves of *Salix miyabeana* (cultivar SX64) plants spread with doses of increased (0, 1.4, 2.1, and 2.8 kg a.e ha^-1^) rates of the glyphosate based herbicide (Factor^®^ 540).** Values are means ± SE of three replicates.

Plants treated with herbicide showed higher activity of all evaluated antioxidant enzymes after 6 h in relation to control (*P* < 0.05; **Figure 4**). We found that: (1) SOD and APX activities were higher in herbicide-treated plants up to 24 h (*P* < 0.0001), and then were reduced for the following exposure times (*P* < 0.0001); (2) CAT activity was always higher in plants treated with herbicide (*P* < 0.0001); (3) similar to SOD and APX, GPX activity was also reduced in herbicide treated plants at 48 and 72 h of exposure (*P* < 0.0001); (4) GR activity was higher in herbicide treated plants up to 48 h of exposure (*P* < 0.05). Regarding ascorbate pool (**Figure 5**) we found that, in relation to control: (1) total ascorbate concentrations (AsA + DHA) were higher in herbicide-treated plants up to 24 h of exposure, and then were reduced for the following exposure times (*P* < 0.0001); (2) the concentrations of the ascorbate reduced form (AsA) were greater in control plants up to 24 h, did not differ between treatments at 48 h and was increased in herbicide treated plants for 72 h (*P* < 0.0001); (3) the concentrations of oxidized form of ascorbate (DHA) were greater in herbicide treated plants up to 24 h and were reduced for the following exposure duration (*P* < 0.0001); (4) the AsA/DHA ratio was lower in 6 and 24 h treated plants compared to control, but was higher for the following treatment times (*P* < 0.0001).

## Discussion

**FIGURE 6:**
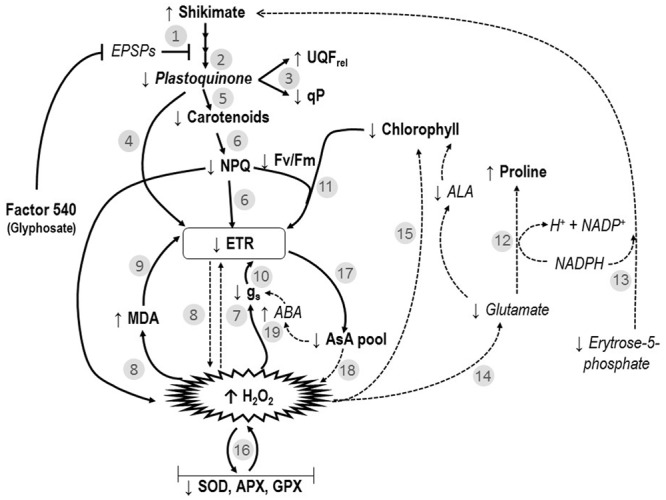
**Interconnected model of the effects of the glyphosate-based-herbicide (Factor^®^ 540) on shikimate pathway, photosynthesis and oxidative markers of willow plants.** Numbers refer to the ones mentioned in the discussion. ABA, abscisic acid; ALA, δ-aminolevulinic acid; APX, ascorbate peroxidase; AsA, ascorbate; EPSPS, 5-enolpyruvylshikimate-3-phosphate synthase; ETR, electron transport rate; *F*_V_/*F*_M_, maximal PSII photochemical efficiency; GPX, glutathione peroxidase; *g*_s_, stomatal conductance; H_2_O_2_, peroxide; *I*_k_, minimum saturating irradiance; MDA, lipid peroxidation; NPQ, non-photochemical quenching; qP, photochemical quenching; SOD, superoxide dismutase; UQF_rel_, the relative unquenched fluorescence. Literature-based information in the models are expressed in italic words and in dotted arrows. While, observed data obtained in the present study are introduced in the model as bold words and non-dotted arrows.

In this study, for the first time, a wide investigation of the impacts of glyphosate-based herbicide on several physiological processes was done. We demonstrated that this type of herbicide affected not only the shikimate pathway, but several physiological processes in willow plants as previously reported by Gomes et al. (2016b). **Figure 6** represents an integrative model interconnecting the studied physiological parameters (in particular, shikimate pathway, photosynthetic processes and oxidative events) affected by exposure to a glyphosate-based herbicide greater than 24 h (48 and 72 h). The various steps of this model are identified throughout the text as **Figure 6**, #1–19.

The glyphosate-based herbicide clearly inhibited the shikimate pathway in willow plants, as demonstrated by the shikimate accumulation (**Figure 3**) and also reported by Huang et al. (2012) and Gomes et al. (2016b). By inhibiting the shikimate pathway (**Figure 6**, #1), the glyphosate-based herbicide may prevent the biosynthesis of several secondary plant compounds (Siehl, 1997), including plastoquinones (**Figure 1**; **Figure 6**, #2). It is known that UQF_rel_ is an indicator of closed PSII reaction centers (RCs) present under continuous illumination (Juneau et al., 2005) and qP represents the proportion of open PSII RCs (Maxwell and Johnson, 2000). Therefore, the higher UQF_rel_ and lower qP in treated plants indicate that the plastoquinone pool, and thus the PSII RCs, were in a more reduced state than in control plants, a consequence of a lower PQ content (**Figure 6**, #2 and 3) and/or less effective PSI. This may, together with the decrease in the *I*_k_ (for doses >1.4 kg a.e ha^-1^), have contributed to the observed lower ETR in treated plants (**Figure 2**; **Figure 6**, #4). Indeed, a lower ability for PSII to deliver electrons to the electron transport chain, leading to PSII saturation at low irradiance, may explain the observed decrease in photosynthesis observed here and in previous studies (Huang et al., 2012; Yanniccari et al., 2012; Gomes et al., 2016b). However, we demonstrated that other effects of the glyphosate-based herbicide have also caused the decrease in the ETR (see below).

The observed increase in the carotenoid concentration after 6 h in the herbicide-treated plants (**Figure 1**) could be related to the concomitant increase in H_2_O_2_ concentration, since it is known that ROS presence can induce carotenogenic responses (Fan et al., 1998). Indeed, by the activation of latent biosynthetic enzymes (such as glutathione transferase and glutathione reductase) or by the expression of genes coding for carotenogenic enzymes, ROS may regulate carotenoid concentration (Aniya and Anders, 1992; Bouvier et al., 1998). Since the maximal PSII photochemical yield (*F*_V_/*F*_M_) is a proxy of the PSII integrity (Walter et al., 2003), *F*_V_/*F*_M_ up to 24 h in treated plants (with the exception of the highest dose; **Figure 2**) indicates that the glyphosate-based herbicide had no effect on the PSII integrity up to 24 h of exposure. Similarly, negative effects of glyphosate in *F*_V_/*F*_M_ of *Lolium perenne* plants were only observed after 3 days of exposure (Yanniccari et al., 2012). This may be the consequence of the increased carotenoid concentration helping to prevent ROS-damages to PSII (Gomes et al., 2013b). Carotenoids are usually involved in the protection of the oxidative damage by the detoxification of oxygen singlets (^1^O_2_) produced by photosynthesis or by enzymatic conversion of other ROS to oxygen singlets (Boussiba, 2000). Although plants exposed to the highest herbicide doses contain high carotenoid concentration, this was not sufficient to prevent oxidative damages to PSII (since we observed lower *F*_V_/*F*_M_ value). These plants also showed higher lipid peroxidation (**Figure 3**) indicating oxidative damages (Gunes et al., 2007), as it was also shown in maize (Sergiev et al., 2006) and rice (Ahsan et al., 2008). However, for exposure times longer than 24 h, plants treated with the glyphosate-based herbicide showed reduced carotenoid concentration (**Figure 1**). This can be a consequence of the inhibition of the shikimate pathway leading to decreased PQ concentration, since plastoquinone is a co-factor of the phytoene desaturase and ζ-carotene desaturase, enzymes involved in the carotenoid biosynthesis pathway (Sandmann et al., 2006). Therefore, decreased plastoquinone concentration will affect directly carotenoid biosynthesis (**Figure 6**, #5). In addition, the decrease in the non-photochemical energy dissipation (NPQ) [one of the mechanisms by which plants can dissipate excess light energy absorbed by PSII light-harvesting complexes in order to minimize the generation of the highly reactive ^1^O_2_ responsible for oxidative damages (Demming-Adams and Adams, 2000)], in plants treated with the glyphosate-based herbicide (**Figure 2**) can be related to the decreased biosynthesis of carotenoids. β-carotene is known to be the precursor of zeaxanthin, the first compound of xanthophyll cycle (Bouvier et al., 1996), and therefore, reduced carotenoid concentration could lead to a lower efficiency of the xanthophyll cycle, reducing plant capacity for photoprotection and thus, leading to increased PSII damages (as shown by reduced *F*_V_/*F*_M_) (**Figure 6**, #6). These decreases in the photosynthetic activity (shown by the decrease in ETR) and in the NPQ may also have contributed to a higher production of ROS (Sergiev et al., 2006; Ahsan et al., 2008; Gomes and Juneau, 2016; Gomes et al., 2016a) due to over-excitation of chlorophylls (**Figure 6**, #7).

As we found in the present study (**Figure 3**; **Figure 6**, #8), increased lipid peroxidation has been previously observed in glyphosate-exposed plants and was related to increased H_2_O_2_ content in plants (Moldes et al., 2008; Miteva et al., 2010; Gomes et al., 2016b). Lipid peroxidation resulting from increased levels of ROS (such as H_2_O_2_) has been shown to affect the integrity of the thylakoid membranes (Richter, 1992), contributing to the noted decrease in ETR (**Figure 6**, #9). Glyphosate was demonstrated to cause depletion of photosynthetic proteins leading to losses of photosynthetic capacity in plants (Diaz Vivancos et al., 2011). However, it has long been recognized that H_2_O_2_ is a potent inhibitor of photosynthesis, since, even at low concentrations, it can inhibit CO_2_ fixation by oxidizing the thiol groups of some essential enzymes of the Calvin cycle (Foyer and Noctor, 2011). We can therefore advance that the observed decrease in photosynthesis (ETR) in presence of glyphosate-based herbicide may also be directly linked to higher H_2_O_2_ concentration (**Figure 5**, #8). Carbon assimilation (and therefore photosynthesis) can also be negatively affected by the decreased stomatal conductance (*g*_s_) (**Figure 1**) in presence of herbicides (Zobiole et al., 2010). Reduced *g*_s_, as also previously reported in *Hordeum vulgare* (barley) and *Lolium perenne* plants exposed to glyphosate (Olesen and Cedergreen, 2010; Yanniccari et al., 2012), can limit photochemistry, resulting in decreased ETR (**Figure 6**, #10). The observed ETR reduction could also be due to the alteration of the integrity of PSII (lower *F*_V_/*F*_M_) (**Figure 2**; **Figure 6**, #11). In addition, the decrease in total chlorophyll concentration in presence of the glyphosate-based herbicide may be responsible for a lower light interception and thus, the noted lower electron transport rate (**Figure 6**, #11). Decreased chlorophyll contents when plants are exposed to herbicide application have been demonstrated previously and have been attributed to an increase chlorophyll degradation or to a decrease in chlorophyll synthesis (Cakmak et al., 2009; Mateos-Naranjo et al., 2009; Huang et al., 2012; Gomes et al., 2016a).

In order to better understand the processes involved in H_2_O_2_ accumulation (and herbicide-induced oxidative damage), we investigated the activity of antioxidant system in treated plants. Increases in proline synthesis is a common protective-response of plants to stress conditions (Hayat et al., 2012). It is important to note, however, that proline can also act as a significant signaling molecule in plant physiological processes, mainly under stress conditions (Hare and Cress, 1997). In the present study, we suggest that the observed proline accumulation in treated plants is associated to oxidative protection, NADP^+^ recovery and shikimate pathway stimulation (**Figure 6**, #12 and #13). As we also observed, proline biosynthesis is commonly stimulated by increased cellular-ROS concentration conditions (Soshinkova et al., 2013). Although proline can be synthesized from ornithine, metabolic labeling studies indicate that, under stress conditions, proline is mainly produced from glutamate (as reviewed by Hare and Cress, 1997). Therefore, the proline accumulation found in our study indicates that this pathway is highly activated (**Figure 6**, #12). A special function of proline in preventing oxidative damage and enhancing tolerance from abiotic oxidative stress has been proposed recently (Soshinkova et al., 2013) and proline accumulation in plants in response to glyphosate exposure was documented (Huang et al., 2012). Due to the loss of feedback control of the shikimate pathway by tyrosine (that regulates the activity of 3-deoxy-D-aravino-heptulosonate-7-phosphate synthase) (Crowley, 2006), the herbicide (glyphosate) led to an unregulated flux of carbon into the shikimate pathway (Siehl, 1997). As a result, there is an increased demand for erythrose-5-phosphate, the substrate of the first reaction of the shikimate pathway. Erythrose-5-phosphate is produced in the oxidative pentose phosphate pathway (OPPP), which is dependent on NAD(P)^+^ availability and inhibited by NADPH (Hare and Cress, 1997). During proline synthesis, NADPH is oxidized, therefore stimulating OPPP. Even a small change in the NAD(P)^+^/NADPH ratio may have a large effect on this redox-sensitive pathway (Hare and Cress, 1997). The oxidation of NADPH during proline synthesis, coupled with the reduction of NADP^+^ during the two oxidative steps of the OPPP, promotes a cycle of changes in NAD(P)^+^/NADPH ratio which stimulates proline biosynthesis, justifying its accumulation during stress (Hare and Cress, 1997). Therefore, upon the glyphosate-based herbicide exposure, the proline accumulation in willow plants could also be linked to the OPPP stimulation for the production of the erythrose-5-phosphate which will be used in shikimate pathway (**Figure 6**, #13). Supporting this hypothesis, Diaz Vivancos et al. (2011) observed decreased NADP/NADPH ratios in leaves of glyphosate-sensitive soybeans upon glyphosate treatment, as a result of the decreases in NADP^+^ pool. As mentioned previously, under stress conditions, proline is mainly produced from glutamate (Hare and Cress, 1997). Glutamate is also required during δ-aminolevulinic acid (ALA; a chlorophyll precursor) biosynthesis through ALA-synthetase and γ,δ-dioxivalerate cycles (Beale, 1978). Therefore, if glutamate was preferentially used for proline biosynthesis (as suggested by proline greater accumulation in treated plants in relation to control; **Figure 3**), a decrease in ALA biosynthesis may be obtained, therefore contributing to the decreased chlorophyll concentration observed in treated plants (**Figure 6**, #14). As suggested by Gomes et al. (2016a), the decrease in chlorophyll concentration may also be due to its degradation by increased ROS content (**Figure 6**, #15).

Even though treated plants showed increased activities of antioxidant enzymes after 6 h exposure, they were not able to prevent both peroxide accumulation and lipid peroxidation, indicating a clear deleterious effect of the glyphosate-based herbicide through oxidative burst. Moreover, the strong inhibition of SOD, APX, and GPX activities observed in plants exposed to herbicide after 48 h (**Figure 4**) can be related to the increased H_2_O_2_ and decreased ETR also observed in these plants. SOD is the first defense enzyme against oxidative stress (Pompeu et al., 2008) and is closely related to stress resistance in plants (Song et al., 2006). Indeed, this enzyme was involved in the PSII protection against the effects of prooxidant herbicides, limiting carbon dioxide and photoinhibitory conditions (Foyer et al., 1994; Arisi et al., 1998). The observed decrease in SOD activity (**Figure 4**) can therefore contribute to the herbicide-deleterious effects on photosynthesis in willow plants. We also demonstrated the key role of APX and GPX to prevent H_2_O_2_ accumulation in willow plants since: (1) decreased activities of both enzymes were related to increased H_2_O_2_ concentration in leaves; (2) even if treated plants shown higher CAT activity, it was not able to prevent H_2_O_2_ accumulation. The importance of APX and GR in avoiding oxidative stress has also been observed in metal(loid) treated plants (Chaoui et al., 1997; Gomes et al., 2013a,b) and the inactivation/degeneration of these enzymes has been related to increased H_2_O_2_ concentrations and oxidative damages to plants (Gomes et al., 2013b). When H_2_O_2_ accumulation exceeded the tolerance limit of plants, enzymatic systems are prone to protein carbonylation–an irreversible oxidative process in which the side chains of Lys, Arg, Pro, and Thr are converted to aldehyde or keto groups (Sohal et al., 2002), which may have been occurring in willow plants exposed to the studied herbicide (**Figure 6**, #16).

We also observed an interesting response of GR activity at 48 and 72 h, since its activity was not significantly decreased by the glyphosate-based herbicide exposure. GR is linked to APX and GPX activity by the glutathione-ascorbate cycle (Foyer and Noctor, 2011). However, as mentioned, the GR activity did not follow APX and GPX patterns upon the herbicide exposure. The maintenance of GR activity in treated plants indicates that APX and GPX activities were not limited by substrate availability, reinforcing that the proposed oxidative damage (protein carbonylation) of the enzymes could be responsible for their degeneration. We may hypothesize that, similarly to the proline production, the higher NADP(H)-dependent-GR activity can favor OPPP and contribute as a source of NADP^+^ for photochemistry.

In addition to being the substrate for APX, ascorbate is an important antioxidant component of the cellular redox potential and its activity is linked to ascorbate-glutathione metabolic cycle (Foyer and Noctor, 2011). In the present study, we found a link between the reduced form of ascorbate (AsA) and the APX activity. Indeed, up to 24 h, treated plants showed higher APX activity concomitantly to the reduced AsA concentration in their leaves; similarly, decreased APX activity for the following treatment periods was related to the increased AsA content. On the other hand, the contrary was observed for the oxidized form of ascorbate (DHA). The accumulation of AsA, as noted by the increased AsA/DHA ratio in treated plants, shows that the DHA has been effectively recycled to AsA by ascorbate-glutathione cycle. We also observed that total ascorbate concentrations (AsA + DHA) were reduced in herbicide treated plants (**Figure 5**). It is known that ascorbate concentration and ETR are closely linked, as the light-dependent stimulation of ascorbate biosynthesis requires photosynthetic electron transport activity (Yabuta et al., 2007). Thus, reduced ETR in treated plants could explain the observed reduction in ascorbate pool (**Figure 6**, #17). Low ascorbate pool favors the increase in both ROS (**Figure 6**, #18) and abscisic acid (ABA), leading to an increase in signal transduction through ROS-mediated and ABA-dependent signaling cascades (Foyer and Noctor, 2011). Among others, the interactive effect of ROS and ABA in stomatal movement is well studied, with increased ROS and ABA content inducing stomatal closure (Gomes et al., 2014a). This mechanism can also be related to the observed herbicide-induced decreases in *g*_s_ (**Figure 6**, #19).

As expected, the primary target site of the studied glyphosate-based-herbicide (Factor^®^ 540) on willow plants is the shikimate pathway. We demonstrated, for the first time, that on top of the alteration of this primary target site, this herbicide induces a series of interconnected events that leads to decreased photosynthetic activity in willow plants. Furthermore, we showed that the herbicide-deleterious effects on photosynthesis are strongly related to herbicide-induced oxidative stress, and that reduction of photosynthesis may amplify the observed effect by inducing ROS production. Our results evidenced that as for photosynthesis-target herbicides, which trigger ROS production and oxidative stress, glyphosate herbicidal effect may be related to induction of ROS accumulation. The inhibition of shikimate pathway may induce changes in redox status with important consequences in leaf metabolism, mainly on photosynthesis. Glyphosate tolerance in plants, for instance, have been related to the ability of plants to deal with ROS accumulation through the activation of antioxidant systems (Maroli et al., 2015). However, since photosynthetic processes of GR plants have been shown to be affected by glyphosate-based herbicides (Zobiole et al., 2010, 2011, 2012), glyphosate may target other cellular sites, inducing ROS formation, for example, mitochondrial electron chain, as proposed by Gomes and Juneau (2016). Although ROS formation may also be produced in the mitochondria, our model fits with several results presented in the literature about the effects of glyphosate in sensitive plants, highlighting the role of ROS induction in this herbicidal mechanism of action.

## Author Contributions

MG, SL performed the experiments; MG, MLa, and PJ designed the experiments; MG and PJ wrote the paper; LH-E and MLu gave technical support and conceptual advice.

## Conflict of Interest Statement

The authors declare that the research was conducted in the absence of any commercial or financial relationships that could be construed as a potential conflict of interest.

## Abbreviations

APX: ascorbate peroxidase
AsA: reduced ascorbate
CAT: catalase
EPSPS: 5-enolpyruvylshikimate-3-phosphate synthase
DHA: dehydroascorbate – oxidized ascorbate
ETR_max_: maximum electron transport rate
*F*_V_/*F*_M_: maximal photochemical efficiency of PSII
GPX: glutathione peroxidase
GR: glutathione reductase
*g*_s_: stomatal conductance
H_2_O_2_: hydrogen peroxide
*I*_k_: minimum saturating irradiance
MDA: malondialdehyde – lipid peroxidation
NPQ: non-photochemical quenching
PQ: plastoquinone
PS: photosystem
qP: photochemical quenching
RLC: rapid light curve
ROS: reactive oxygen species
SOD: superoxide dismutase
UQF_rel_: relative unquenched fluorescence
