# Supplementary material for: Glyphosate-Dependent Inhibition of Photosynthesis in Willow
Source: Front Plant Sci. 2017 Feb 17;8:207. doi: 10.3389/fpls.2017.00207 (PMC5314154; doi:10.3389/fpls.2017.00207)
Supplement: Supplementary file 1 [file Table_1.DOCX]

**Table 1S.** MANOVA repeated-measures for the effects of glyphosate treatment (kg a.e ha^-1^) and time of exposure (hours) on total chlorophyll and carotenoid concentrations (µg g^-1^ FW), plastoquinone pool (PQ - nmol g^-1^ FW), stomatal conductance (*g_s_* - mmol m^-2^s^-1^), maximum electron transport rate (ETR_max_ - µmol electrons m^-2^ s^-1^), minimum saturating irradiance (I_k_ - µmol photons m^-2^ s^-1^ ), photochemical quenching (qP), the relative unquenched fluorescence (UQF_rel_), non-photochemical quenching (NPQ) and the photochemical efficiency of PSII (Fv/Fm) in leaves of *Salix miyabeana* (cultivar SX64). D.F, degrees of freedom; *significant.

| Source of Variation | D.F | Chl | Carot | PQ | g_s_ | ETR_max_ | Ik | qP | UQF_rel_ | NPQ | Fv/Fm |
| --- | --- | --- | --- | --- | --- | --- | --- | --- | --- | --- | --- |
| Glyphosate | 3 | <0.0001^*^ | <0.05^*^ | <0.001^*^ | <0.01^*^ | <0.0001^*^ | <0.0001^*^ | <0.0001^*^ | <0.0001^*^ | <0.001^*^ | <0.0001^*^ |
| Time | 3 | <0.0001^*^ | <0.0001^*^ | <0.0001^*^ | <0.001^*^ | <0.0001^*^ | <0.05^*^ | <0.0001^*^ | <0.05^*^ | <0.0001^*^ | <0.001^*^ |
| Glyphosate x Time | 9 | 0.0688 | <0.0001^*^ | 0.1641 | 0.1518 | <0.001^*^ | <0.001^*^ | <0.0001^*^ | <0.05^*^ | <0.05^*^ | <0.001^*^ |
