# Supplementary material for: Glyphosate-Dependent Inhibition of Photosynthesis in Willow
Source: Front Plant Sci. 2017 Feb 17;8:207. doi: 10.3389/fpls.2017.00207 (PMC5314154; doi:10.3389/fpls.2017.00207)
Supplement: Supplementary file 2 [file Table_2.DOCX]

**Table 2S.** MANOVA repeated-measures for the effects of glyphosate treatment (kg a.e ha^-1^) and time of exposure (hours) on shikimate (µmol g^-1^ FW), proline (µmol g^-1^ FW), H_2_O_2_ (mmol g^-1^ FW), MDA (nmol g^-1^ FW) concentration; activity of SOD (U mg^-1^ protein), CAT (µmol H_2_O_2_ min^-1^ g^-1^ protein), APX (µmol ascorbate m^-1^ mg^-1^ protein), GPX (µmol glutathione m^-1^ mg^-1^ protein) and GR (µmol NADPH min^-1^ mg^-1^ protein); concentration of total (AsA + DHA–nmol g^-1^ FW), reduced (AsA - nmol g^-1^ FW) and oxidized (DHA - nmol g^-1^ FW) form of ascorbate and Asa/DHA ratios in leaves of *Salix miyabeana* (cultivar SX64). D.F, degrees of freedom; *significant.

| Source of Variation | D.F | Shikimate | Proline | H_2_O_2_ | MDA | SOD | CAT | APX | GPX | GR | AsA + DHA | AsA | DHA | Asa/DHA |
| --- | --- | --- | --- | --- | --- | --- | --- | --- | --- | --- | --- | --- | --- | --- |
| Glyphosate | 3 | <0.0001^*^ | <0.0001^*^ | <0.001^*^ | <0.0001^*^ | <0.05^*^ | <0.0001^*^ | 0.5778 | <0.001^*^ | <0.0001^*^ | <0.001^*^ | <0.05^*^ | <0.05^*^ | <0.05^*^ |
| Time | 3 | <0.001^*^ | <0.01^*^ | <0.01^*^ | <0.05^*^ | <0.0001^*^ | <0.0001^*^ | <0.0001^*^ | <0.0001^*^ | <0.05^*^ | <0.0001^*^ | <0.0001^*^ | <0.0001^*^ | <0.05^*^ |
| Glyphosate x Time | 9 | <0.0001^*^ | <0.01^*^ | 0.0811 | <0.0001^*^ | <0.001^*^ | 0.08 | <0.0001^*^ | <0.0001^*^ | <0.01^*^ | <0.0001^*^ | <0.0001^*^ | <0.0001^*^ | <0.0001^*^ |
